# Heparanase promotes myeloma progression by inducing mesenchymal features and motility of myeloma cells

**DOI:** 10.18632/oncotarget.7170

**Published:** 2016-02-03

**Authors:** Juan Li, Qianying Pan, Patrick D. Rowan, Timothy N. Trotter, Deniz Peker, Kellie M. Regal, Amjad Javed, Larry J. Suva, Yang Yang

**Affiliations:** ^1^ Department of Pathology, University of Alabama at Birmingham, Birmingham, AL, USA; ^2^ Department of Hematology, the First Affiliated Hospital of Sun Yat-Sen University, Guangdong, China; ^3^ Comprehensive Cancer Center and the Center for Metabolic Bone Disease, University of Alabama at Birmingham, Birmingham, AL, USA; ^4^ Department of Oral and Maxillofacial Surgery, School of Dentistry, University of Alabama at Birmingham, Birmingham, AL, USA; ^5^ Department of Veterinary Physiology and Pharmacology, College of Veterinary Medicine and Biomedical Sciences, Texas A & M University, College Station, TX, USA

**Keywords:** multiple myeloma, bone dissemination, heparanase, mesenchymal marker, tumor microenvironment

## Abstract

Bone dissemination and bone disease occur in approximately 80% of patients with multiple myeloma (MM) and are a major cause of patient mortality. We previously demonstrated that MM cell-derived heparanase (HPSE) is a major driver of MM dissemination to and progression in new bone sites. However the mechanism(s) by which HPSE promotes MM progression remains unclear. In the present study, we investigated the involvement of mesenchymal features in HPSE-promoted MM progression in bone. Using a combination of molecular, biochemical, cellular, and *in vivo* approaches, we demonstrated that (1) HPSE enhanced the expression of mesenchymal markers in both MM and vascular endothelial cells; (2) HPSE expression in patient myeloma cells positively correlated with the expression of the mesenchymal markers vimentin and fibronectin. Additional mechanistic studies revealed that the enhanced mesenchymal-like phenotype induced by HPSE in MM cells is due, at least in part, to the stimulation of the ERK signaling pathway. Finally, knockdown of vimentin in HPSE expressing MM cells resulted in significantly attenuated MM cell dissemination and tumor growth *in vivo*. Collectively, these data demonstrate that the mesenchymal features induced by HPSE in MM cells contribute to enhanced tumor cell motility and bone-dissemination.

## INTRODUCTION

Multiple myeloma (MM) is a malignant plasma cell disorder that accounts for approximately 10% of all hematologic cancers [1]. Despite the advances made with therapies in the last 20 years, MM still remains largely incurable [2]. Heparanase (HPSE), an enzyme that cleaves the heparan sulfate chains of proteoglycans in the tumor microenvironment [3, 4], has been shown by us and others to promote tumor growth, angiogenesis and correlate with bone metastasis of a variety of cancer cells, including MM [5–10]. However, the mechanisms by which HPSE promotes MM cell dissemination and progression in bone remain unclear.

Recently, studies demonstrated that enhanced expression of mesenchymal markers and acquirement of a mesenchymal phenotype (so called the epithelial-to-mesenchymal transition (EMT)) play a critical role in the metastases of solid tumors [11–13]. However, whether mesenchymal features are induced in aggressive MM cells (for example, heparanase-highly-expressed MM cells) and play a role in MM cell dissemination have not been investigated. In the present study, using *in vivo* and *in vitro* tools, we demonstrate that HPSE induces mesenchymal feature in MM cells, which contributes to tumor cell motility and bone-dissemination.

## RESULTS

### HPSE enhances the expression of mesenchymal markers in both myeloma cells and vascular endothelial cells

**Figure 1:**
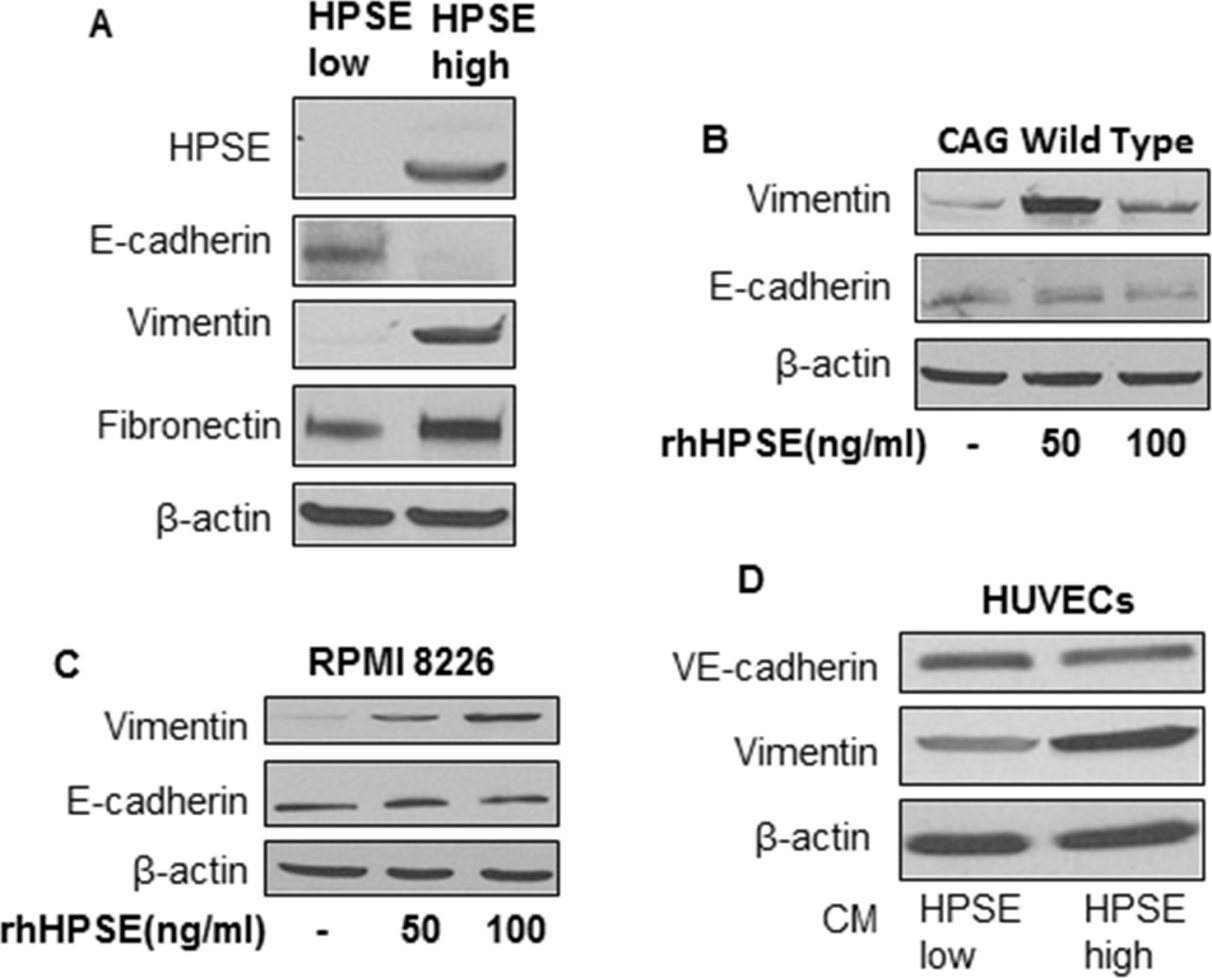
HPSE induces a mesenchymal phenotype in myeloma cells and vascular endothelial cells (**A**) Total cellular protein was isolated from HPSE-low or HPSE–high CAG MM cells and Western blotting was performed for heparanase and EMT-associated protein expression (E-cadherin, vimentin and fibronectin), β-actin is a loading control. (**B**) CAG wild type and (**C**) RPMI 8226 cellswere cultured in the absence or presence of recombinant human HPSE (50 ng/ml or 100 ng/ml) for 48 hrs. Cell lysates were analyzed by Western blot for vimentin, E-cadherin and β-actin protein expression. (**D**) HUVECs (human umbilical vein endothelial cells) were cultured in the conditioned medium of CAG HPSE-low or CAG HPSE-high cells with equal volumes of EGM-2 medium for 72 hrs. Protein was isolated and Western blotting was performed for VE-cadherin, vimentin and β-actin expression.

To assess the impact of HPSE on the expression of epithelial marker E-cadherin and mesenchymal markers vimentin and fibronectin in MM cells, cellular protein was isolated from HPSE-low (human MM CAG cells transfected with empty vector) and HPSE-high (CAG cells transfected with human HPSE cDNA) MM cells [9, 14], and E-cadherin, Vimentin and fibronectin expression was examined by Western blotting. The results revealed a significantly decreased E-cadherin and increased vimentin and fibronectin expression in HPSE-high CAG cells, compared to those in HPSE-low CAG cells (Figure 1A). To further determine the relationship between HPSE expression and the expression of epithelial marker and mesenchymal marker in different MM cell lines, wild-type CAG and RPMI 8226 human MM cell lines were cultured in the absence or presence of recombinant human HPSE (rhHPSE) for 48 hrs, E-cadherin and vimentin expression were assessed by Western blot. Similar to HPSE transfected cells (HPSE-high cells), the addition of rhHPSE resulted in significantly enhanced vimentin expression in both wild-type CAG and RPMI 8226 myeloma cell lines, however E-cadherin expression was only slightly decreased (Figure 1B and 1C).

We have shown previously that HPSE promotes the motility and angiogenic potential in endothelial cells [15]. To determine whether HPSE also stimulates endothelial cells to express higher levels of mesenchymal marker, conditioned medium (CM) harvested from CAG HPSE-low or HPSE-high MM cells was added to cultures of human umbilical vein endothelial cells (HUVECs) in a 1:1 ratio with standard HUVEC medium. After 72 hr, HUVECs were lysed and the levels of the endothelial marker VE-cadherin and mesenchymal marker vimentin were evaluated by Western blot. As shown in Figure 1D, VE-cadherin expression was slightly inhibited and vimentin expression remarkably increased in the HUVEC cells treated with the CM of CAG HPSE-high cells, compared to the cells treated with CAG HPSE-low CM. Taken together, these data demonstrate that HPSE induces mesenchymal phenotype in both MM cells and endothelial cells, which may contribute to enhanced MM dissemination and angiogenesis. However, HPSE seems having limited influence in the expression of epithelial/endothelial markers.

### Heparanase induces a mesenchymal phenotype in MM cells *in vivo* and this process is blocked by HPSE inhibitor SST0001

**Figure 2:**
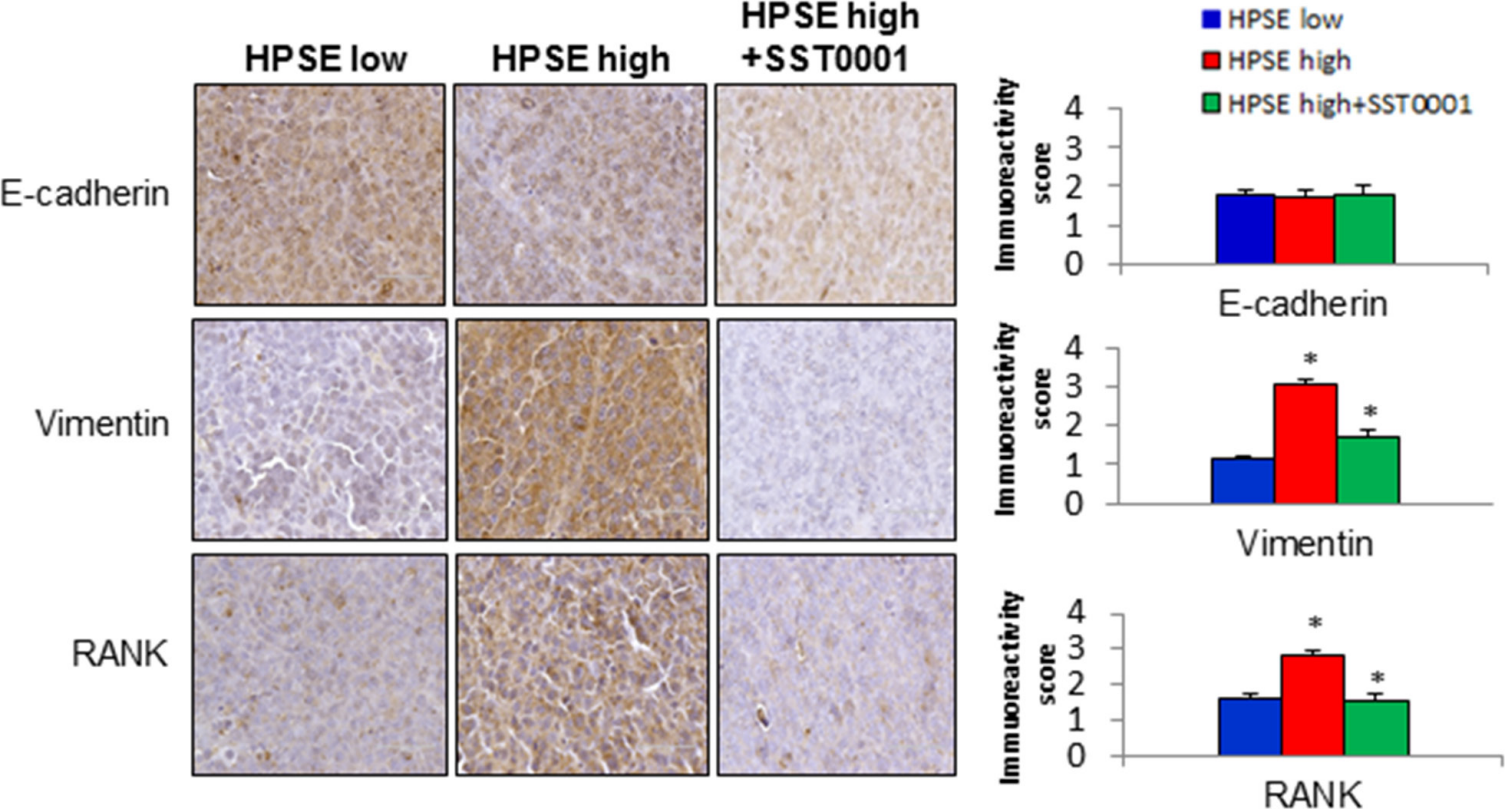
HPSE-High MM tumors exhibit a strong mesenchymal phenotype that can be reversed by HPSE inhibition Tumor xenografts formed by CAG HPSE-low or CAG HPSE-high cells, or CAG HPSE-high + the HPSE-specific inhibitor, SST0001, (*n* = 8 per group) were harvested and stained for human E-cadherin, vimentin and RANK expression. Photomicrographs (original magnification 400×) are shown (left). Immunostaining density for E-cadherin, vimentin, and RANKin tumors from HPSE-low, HPSE-high, and HPSE-high + SST0001 was evaluated in 5 random areas of each tumor section at 200× and was scored as 0+ (no staining), 1+ (weak staining), 2+ (moderate staining), 3+ (strong staining), 4+ (very strong staining) (right). Bar graphs show the mean + SEM. *p < 0.05, CAG HPSE-low vs. CAG HPSE-high or CAG HPSE-high vs. CAG HPSE-high + SST0001.

We have demonstrated that MM tumors formed from CAG cells expressing high levels of heparanase grow and progress to bone much more readily than CAG tumors expressing low levels of heparanase [9] and that the HPSE inhibitor SST0001 inhibits tumor growth in MM animal models [16]. To determine whether HPSE promotes the expression of mesenchymal markers *in vivo*, tumors were harvested from SCID mice bearing CAG HPSE-low or CAG HPSE-high tumors in the presence or absence of treatment with SST0001 (30 mg/kg/day, 28 days) [16]. Formalin-fixed tumors were sectioned for immunohistochemical staining with antibodies against the epithelial marker E-cadherin and the mesenchymal marker vimentin. Analysis of the specific staining showed slightly decreased E-cadherin and significantly increased vimentin expression in the tumors formed by CAG HPSE-high cells, compared to tumors formed by CAG HPSE-low cells (Figure 2). In addition, expression of the mesenchymal marker receptor activator of NF-kB (RANK) [17] was also significantly higher in the tumors formed by CAG HPSE-high cells (Figure 2). In contrast, significantly decreased expression of vimentin and RANK was observed in the CAG HPSE-high tumors treated with SST0001 (Figure 2).

### Heparanase expression positively correlates with the expression of mesenchymal markers in myeloma cells of MM patients

**Figure 3:**
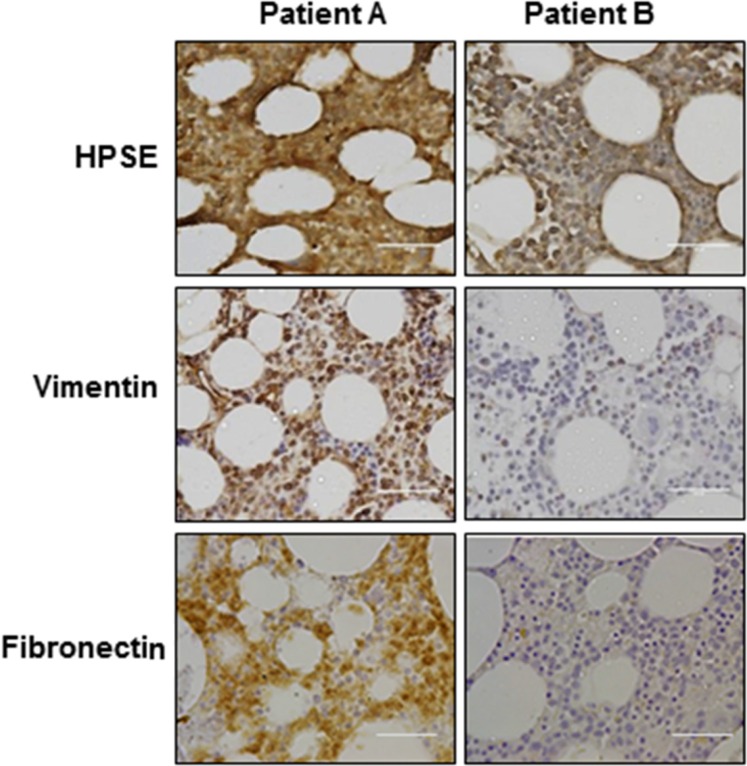
Heparanase expression is positively correlated with expression of vimentin and fibronectin in myeloma patient cells Heparanase (HPSE), vimentin and fibronectin staining of two representative myeloma patient bone marrow biopsy specimens. Complete patient analysis is shown in Supplementary Tables 1 and 2. PatientA: HPSE expression is high in MM cells, and vimentin, fibronectin expression are also high. Patient B: HPSE expression is low in MM cells, and vimentin, fibronectin expression are also low. Original magnification, 400×. Bar = 100 um.

The correlated expression of HPSE and multiple mesenchymal markers demonstrated *in vitro* and *in vivo* led us to investigate whether HPSE drives a mesenchymal phenotype in myeloma patients. Specific immunohistochemical staining for HPSE and mesenchymal marker vimentin was performed on 35 newly diagnosed, treatment naïve myeloma patient bone marrow core biopsy specimens. The staining density of MM cells was scored as described previously [18, 19]. We identified a significantly positive correlation between HPSE and vimentin expression in MM cells (*r*_s_ = 0.414, *p* = 0.014) (Figure 3 and Supplementary Tables 1). In a later setting, 14 bone marrow core biopsy specimens from newly diagnosed myeloma patient were stained for HPSE and the mesenchymal marker fibronectin. A positive and significant correlation between HPSE and fibronectin expression by MM cells was found (*r*_s_ = 0.55, *p* < 0.05) (Figure 3 and Supplementary Tables 2). These data confirmed that HPSE induces mesenchymal-like features in MM cells.

### Blocking ERK signaling pathway reverses vimentin expression in myeloma cells

**Figure 4:**
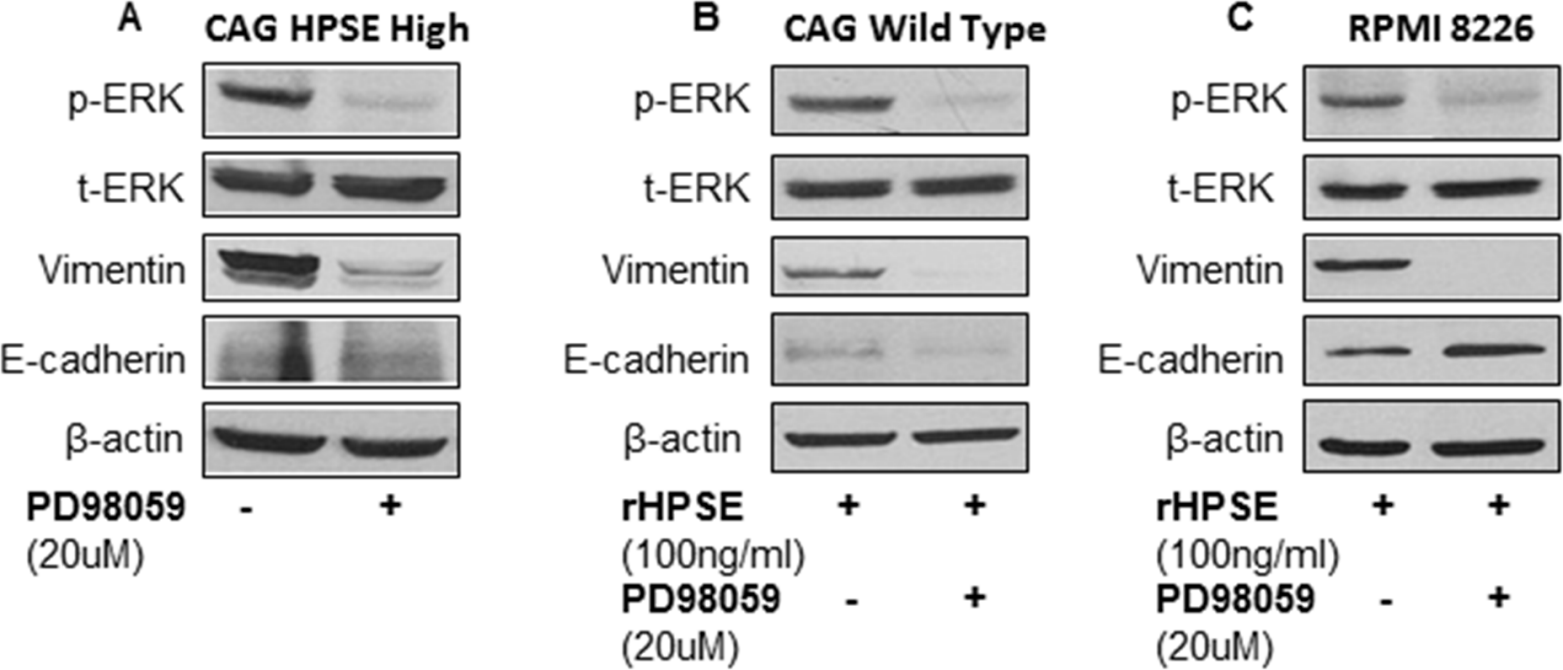
ERK signaling inhibitor reverses vimentin expression in myeloma cells (**A**) CAG HPSE-high cells were treated with or without the ERK inhibitor PD98059 (20 uM) for 48 hrs. Cells were lysed and Western blotting performed for the expression of phosphorylated-ERK (p-ERK), total-ERK (t-ERK), vimentin, E-cadherin, and β-actin. (**B**) CAG wild type and (**C**) RPMI8226 cells were cultured in the presence of rhHPSE (100 ng/ml) with or without ERK inhibitor, PD98059 (20 uM) for 48 hrs. Total protein was isolated from the cells and analyzed by Western blot using antibodies against p-ERK, t-ERK, Vimentin, E-cadherin, and β-actin.

The ERK pathway is required for the progression of mesenchymal transition in a variety of tumor cells [20, 21]. We have previously demonstrated that ERK signaling was elevated by HPSE in MM cells [16, 22, 23]. To identify the intracellular signaling pathway involved in HPSE-promoted expression of mesenchymal markers, CAG HPSE-high cells were treated with or without ERK signaling pathway inhibitor PD98059 (20 uM) for 48 hrs. Western blotting revealed a significant inhibition of vimentin expression following inhibition of ERK activity; however, the change of E-cadherin expression was not obvious (Figure 4A). In addition, decreased vimentin expression was also observed in CAG and RPMI 8226 MM cells treated with both rhHPSE and PD98059, compared to the cells treated with rhHPSE alone (Figure 4B and 4C). These results suggest that the activation of ERK signaling is involved in the HPSE-enhanced mesenchymal phenotype of MM cells.

### Knockdown of vimentin in CAG HPSE-high MM cells inhibits tumor growth and homing to bone *in vivo*

**Figure 5:**
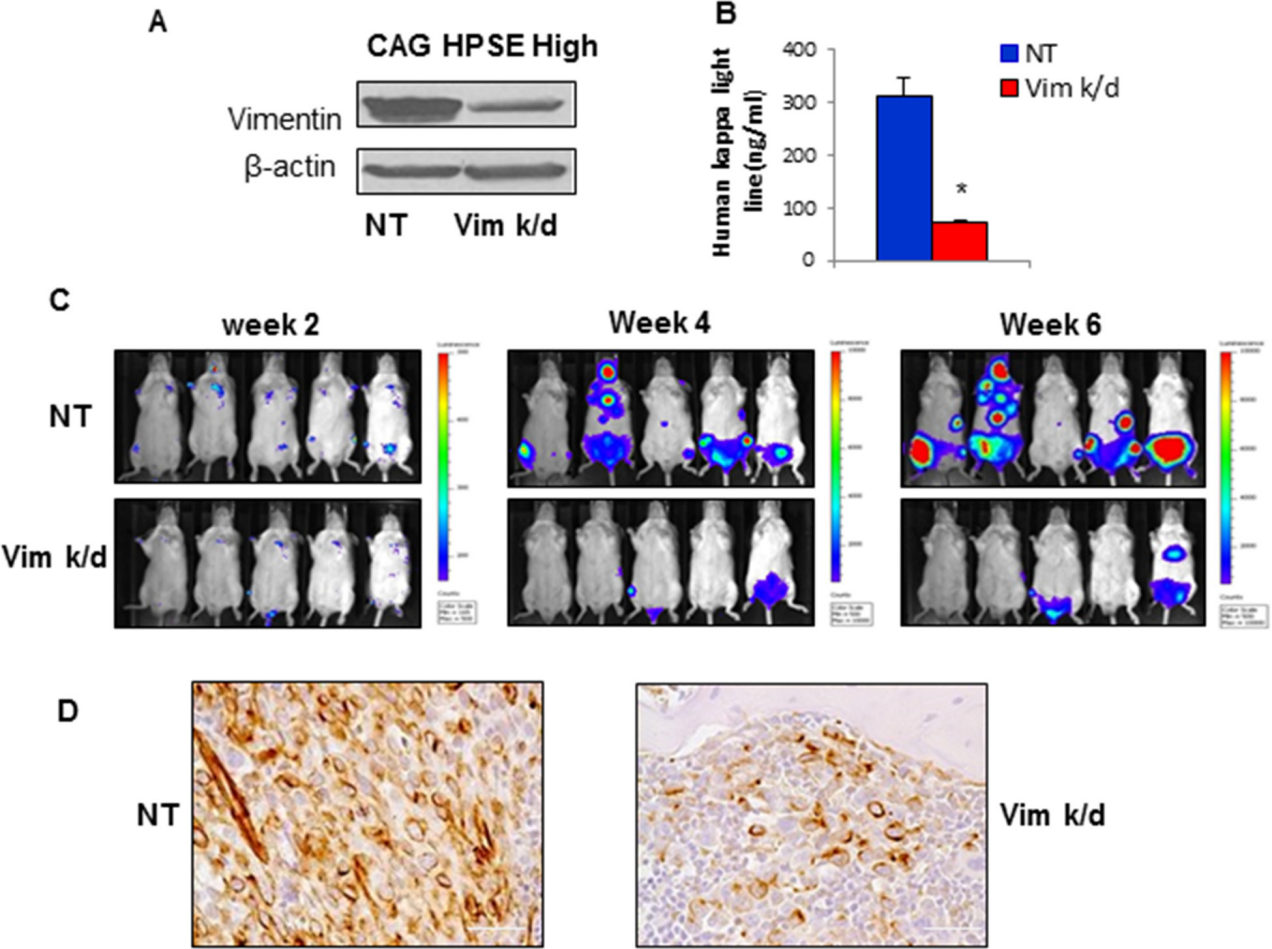
Vimentin knockdown in CAG HPSE-high MM cells inhibits tumor growth and bone homing *in vivo* (**A**). Reduced vimentin expression in CAG HPSE-high vimentin k/d (Vim k/d) cells compared to mock transfected control cells (NT). (**B**) Levels of human immunoglobulin kappa light chain in murine serum (an indicator of whole animal tumor burden) were measured 6 weeks after tumor cell inoculation in mice bearing NT and Vim k/d CAG HPSE-high tumors. Data is presented asmean ± SEM. *p < 0.05. (**C**) Representative bioluminescent images (5 animals per group) 2, 4, and 6 weeks after tumor cell injection. Marked suppression of tumor progression in Vim k/d tumors is evident. (**D**) Vimentin staining of representative tumors formed by NT and Vim k/d CAG HPSE-high cells. Original magnification, 400×. Bar = 100 um.

To confirm the role of mesenchymal marker vimentin in MM progression, we knocked down vimentin expression using vimentin shRNA in CAG HPSE-high MM cells (Vim k/d cells). Control CAG HPSE-high cells were infected with a non-targeted shRNA (NT cells) (Figure 5A). The NT control or Vim k/d cells expressing luciferase were then injected intravenously via tail vein into SCID mice (*n* = 5) and the animals were monitored for disease progression by bi-weekly bioluminescence imaging. Two weeks after MM cells injection, 4 out of 5 mice injected with NT control cells formed visible tumors in bone. In contrast, only 1 of 5 mice injected with Vim k/d cells showed an evidence of tumor in bone (Figure 5C). Six weeks after MM cell injection, bioluminescence imaging revealed that all mice in the NT group had bone tumors, whereas only 2 of 5 mice injected with the Vim k/d cells had visible tumors in bone. However, Vim k/d tumors were significantly smaller than NT tumors (Figure 5C). In addition, the levels of human immunoglobulin kappa light chain (a soluble marker of CAG cells) in murine sera, measured by ELISA [9], demonstrated a significantly lower tumor burden in the mice injected Vim k/d cells than the mice injected NT control cells (Figure 5B). After sacrificing the mice, the hindlimbs from control and Vim k/d mice were sectioned. H & E staining confirmed that all five control mice had tumor in bone, but only two out-of-five mice in the Vim k/d group had tumors that were of much smaller size than those in the control mice (Data not shown). Human vimentin staining demonstrated that vimentin expression in Vim k/d tumor is significantly less than in control tumors, demonstrating that the Vim k/d CAG cells kept the vimentin-k/d phenotype *in vivo* (Figure 5D).

### Heparanase promotes MM cell spreading and migration through vimentin

**Figure 6:**
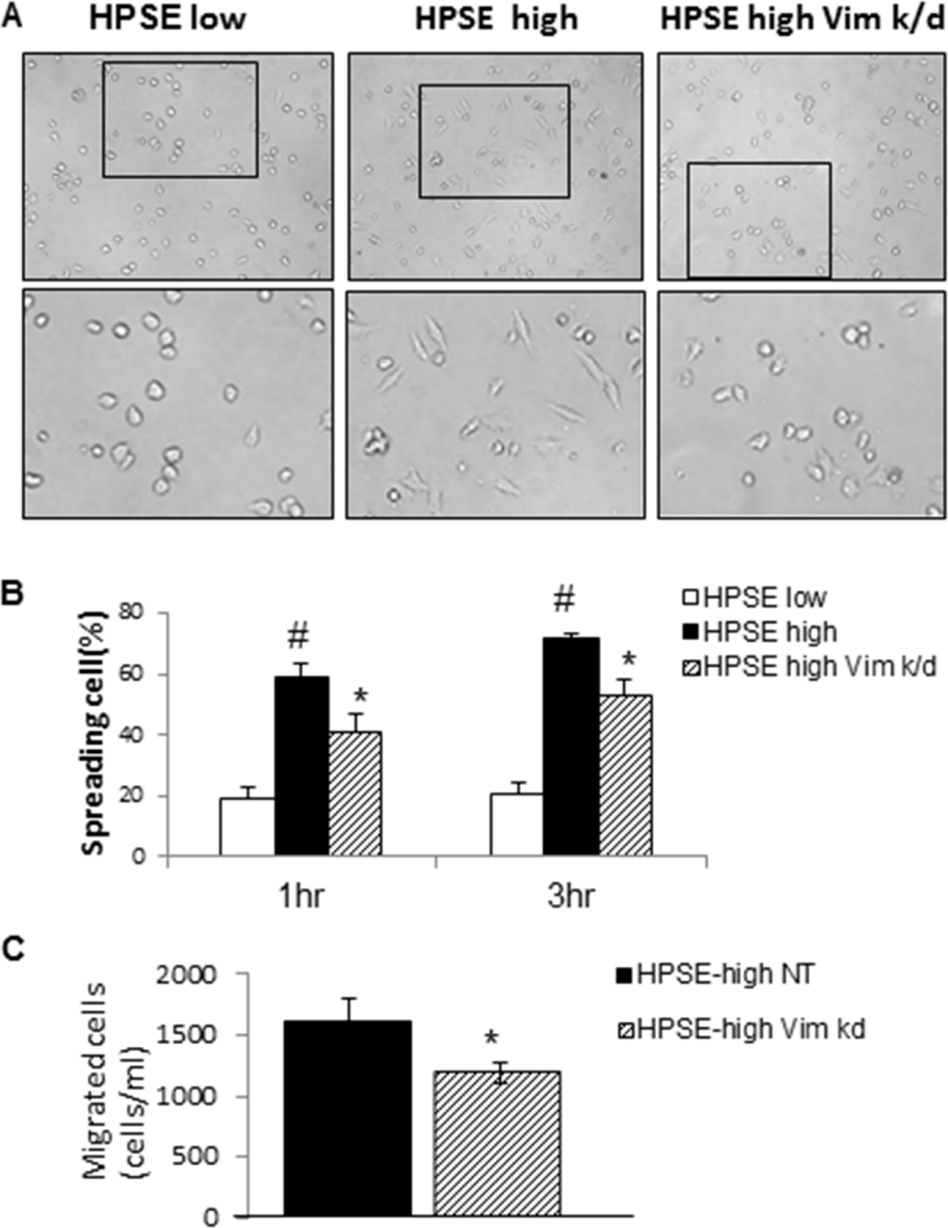
Heparanase induces CAG MM cell spreading that is inhibited by knockdown of vimentin (**A**) HPSE-low, HPSE-high and HPSE-high vimentin k/d cells were seeded on plates pre-coated with fibronectin. Representative photomicrographs 200× (upper) and upper boxed region 400× (lower) are shown 3 hrs after cell seeding. (**B**) The percentage of spreading cells among the total cells in the entire area is shown. Measurements were made in 5 random areas for each cell line at each time point (1 and 3 hours post seeding). Each bar represents the Mean ± SD. #*p* < 0.05, compared to HPSE low; *p < 0.05, compared to HPSE high. (**C**) Migration assay using HPSE-high NT and HPSE-high vimentin k/d cells. Each bar represents the Mean ± SD.*p < 0.05, compared to HPSE-high NT.

Cell spreading is a key step of cancer metastasis [24]. To examine the influence of vimentin on HPSE-induced MM cell spreading, the spreading ability of HPSE-low, HPSE-high and HPSE-high/Vim k/d cells were compared using a cell-spreading assay on fibronectin. As shown in Figure 6, HPSE-high MM cells displayed significantly enhanced spreading, compared to HPSE-low MM cells, and vimentin knockdown significantly inhibited HPSE-high MM cell spreading on fibronectin (Figure 6A–6B).

Our previous experiments have demonstrated that enhanced heparanase expression in MM cells promotes MM cell migration [9, 22]. To determine whether heparanase-promoted MM cell migration relies on vimentin expression, the same migration assay was performed using HPSE-high and HPSE-high/Vim k/d cells. As shown in Figure 6C, vimentin knockdown significantly inhibited MM migration. However, no significant differences in cell proliferation were observed between HPSE-high and HPSE-high/Vim k/d cells.

## DISCUSSION

Multiple myeloma is a clonal plasma cell disorder characterized by the presence of multiple lytic bone lesions, suggesting that there is continuous trafficking of tumor cells to multiple sites within the bone marrow [25–27]. The metastasis of solid tumors involves a complex and interrelated series of steps that eventually culminate in tumor cell progression at a specific metastatic site [28]. Mesenchymal transition is considered the first step in the metastatic process of epithelial-derived cancers [27]. MM is a hematologic cancer that is not of epithelial-origin. However, MM has a high degree of plasticity with tumor cell phenotypes known to change in response to specific microenvironmental queues [28]. Indeed, one study has shown that hypoxia can lead to the inactivation of E-cadherin and activation of the transcription factors regulating EMT in myeloma cells [29]. In the present study, we demonstrate, through the analysis of multiple MM cell lines and primary myeloma cells from MM patients that myeloma cells do express a number of EMT markers (e.g., E-cadherin, Vimentin, Fibronectin, and RANK) and that the expression of these markers is regulated by HSPE.

HPSE is an enzyme highly expressed in aggressive MM cells and that we and others have shown is a key promoter of MM progression via the remodeling of the tumor microenvironment [9, 14, 18, 19]. In this study, we further demonstrate that HPSE enhances the expression of the mesenchymal markers vimentin, fibronectin and RANK, and consequent motility of MM cells [17, 29, 30]. In addition, *in vitro* and *in vivo* studies show that the specific HPSE inhibitor SST0001 blocks HPSE-induced mesenchymal features. However the expression of the epithelial marker E-cadherin was only slightly decreased in HPSE transfected MM cells or in MM cells cultured with recombinant heparanase treatment. This may be because MM cells are not of epithelial cell origin and have a low basal level of E-cadherin expression. Taken together, these results demonstrate that HPSE actively drives a mesenchymal phenotype that enhances the motility of MM cells, which may contribute to MM spread within local bone sites and dissemination to distant bone sites. These data provide the evidence that suggest a novel mechanism by which HPSE promotes the development of mesenchymal features that drives the aggressive osteolytic phenotype characteristic of MM.

Among the mesenchymal markers upregulated by HPSE, the increased level of vimentin is most apparent. To further ensure that enhanced mesenchymal features are indeed involved in MM progression in bone *in vivo*, we knocked down vimentin expression in CAG-HPSE-high MM cells and injected the cells into SCID mice. We observed that vimentin inhibition significantly prevented HPSE-high myeloma cells from homing to and growing in bone. These *in vivo* data suggest that HPSE may promote MM progression in bone via a novel mechanism perhaps involving an induced mesenchymal phenotype in MM cells. Further mechanistic study to ascertain how vimentin plays a role in HPSE-driven MM dissemination and progression are currently ongoing.

Several signaling pathways are known to be involved in mesenchymal transition in solid tumors, including TGF-β, NF-kB, Wnt, Notch and ERK. [31]. Since we have previously demonstrated that HPSE activates ERK signaling in MM cells [22], the potential role of the ERK pathway in the HPSE-induced mesenchymal feature in myeloma cells was investigated by treating HPSE-high MM cells or MM cells cultured in the presence of rhHPSE with the ERK signaling pathway inhibitor PD98059. These experiments demonstrated that inhibition of ERK signaling significantly inhibited HPSE (both transfected HPSE and rhHPSE) enhanced vimentin expression, suggesting the involvement of the ERK pathway in HPSE-enhanced mesenchymal phenotype in myeloma cells.

We previously demonstrated that MM cell-derived HPSE not only induced an aggressive phenotype in myeloma cells, but also promoted angiogenesis via the secretion of soluble factors, such as HGF, VEGF and soluble syndecan-1, from myeloma cells [10, 14, 32]. To determine whether HPSE promoted angiogenesis through the induction of a mesenchymal phenotype in the host vascular endothelial cells in the tumor microenvironment, HUVECs were cultured in conditioned medium (CM) of HPSE-low or HPSE-high cells and the expression of the mesenchymal marker vimentin was analyzed by Western blot. The results demonstrated that MM cell-derived HPSE stimulated vimentin expression in HUVECs. These data support the hypothesis that HPSE promotes angiogenesis in MM via promoting mesenchymal transition of endothelial cells. Additional studies are currently ongoing to further delineate the mechanistic details of this important finding.

In summary, the findings presented herein demonstrate that mesenchymal features can be induced in MM cells by HPSE, and that this mesenchymal phenotype is responsible, at least in part, for HPSE-promoted MM dissemination and progression in bone. In addition, MM cell-derived HPSE may also promote angiogenesis by inducing a mesenchymal transition in host vascular endothelial cells. Together our studies demonstrate a novel mechanism of HPSE action in MM and emphasize the importance of HPSE in remodeling the bone marrow microenvironment in favor of MM progression.

## MATERIALS AND METHODS

### Cells and reagents

The CAG myeloma cell line was established at the Myeloma Institute for Research and Therapy (Little Rock, AR) as described previously [32]. CAG cells with modified levels of heparanase (HPSE) expression have been previously and extensively characterized: (a) HPSE-low cells prepared by transfection with empty vector and express low level of endogenous heparanase; (b) HPSE-high cells prepared by transfection with vector containing the cDNA for human heparanase [9, 16]. RPMI 8226 human myeloma cell line and human umbilical vein endothelial cells (HUVECs) were purchased from the American Type Culture Collection (Manassas, VA). RPMI 8226 and CAG myeloma cells were cultured in RPMI1640 growth medium supplemented with 10% fetal bovine serum, 1% antibiotic/antimycotic and 1% L-glutamine. HUVECs were cultured in endothelial cell basal medium-2, supplemented with growth supplements and 2% fetal bovine serum (Lonza).

Recombinant human heparanase (rhHPSE) and human HPSE antibodies were kindly provided by Dr. Ralph D. Sanderson (UAB, AL). Human E-cadherin, vimentin, phosphorylated-ERK, total-ERK antibodies were purchased from Cell Signaling (catalog #'s 3195, 5741, 4370 and 4695 respectively). Human Fibronectin and RANK antibodies were from R & D Systems (catalog #'s MAB1918 and MAB6831). Human VE-cadherin antibody was from Santa Cruz (catalog # sc-9989). β-actin antibody (catalog # A5316) and PD98059 ERK inhibitor were from Sigma-Aldrich. SST0001 is a potent inhibitor of heparanase that was produced by Sigma-tau [33].

### Preparation of conditioned medium (CM) of HPSE-low and HPSE-high cells

HPSE-low and HPSE-high myeloma cells were seeded at a concentration of 5 × 10^5^ cells/ml in RPMI1640 medium supplemented with 10% fetal bovine serum and incubated for 48 h at 37°C and 5% CO_2_ in a humidified chamber. Medium conditioned by the cells was collected at the end of the incubation period and centrifuged at 1000 rpm for 5 minutes to remove the cells. The medium was aliquoted and stored at −80°C until further use.

### Cell lysis and western blotting

Total protein was isolated from cultured cells using protein lysis buffer (Thermo). Protein concentration was determined by BCA assay (Thermo). Equal amounts of protein (100 ug) from cell extracts were subjected to 4% to 15% gradient SDS-PAGE gels (Bio-Rad) and subsequently transferred to nitrocellulose membranes. After blocking for 1 h with TBS containing 0.1% Tween20 and 5% non-fat dry milk, membranes were incubated with primary antibodies. Secondary antibody conjugated with horseradish peroxidase (HRP) (GE Healthcare) was used at 1:2000 dilution to detect primary antibodies, and enzymatic signals were visualized by an enhanced chemiluminescence system (Amersham Biosciences).

### Immunohistochemistry

Immunohistochemistry was performed on formalin-fixed, paraffin-embedded tissue sections. Briefly, sections were deparaffinized with xylene and then rehydrated through graded concentrations of ethanol and distilled water. Epitope retrieval was performed by steaming the slides for 20 min in citrate buffer solution. Slides were then washed and quenched with 3% hydrogen peroxide and blocked with 5% BSA in PBS. The sections were probed with primary antibody, washed with PBS and incubated with an appropriate biotin-conjugated secondary antibody (Vector Laboratories). The slides were then incubated with Vectastain ABC reagent (Vector Laboratories) with detection by incubation with 3, 3′-diaminobenzidine (DAB). The sections were counterstained with Harris Hematoxylin and scored in a blinded fashion by two independent readers as described previously [14, 18]. The readers assigned the samples scores of 0 for negative samples to 1+ for least intensely positive to 4+ for most intensely positive.

Paraffin-embedded bone marrow core biopsy specimens from untreated myeloma patients, obtained from the Department of Pathology at UAB, were staining for heparanase, vimentin and fibronectin. The experimental procedures and protocols were approved by the UAB Institutional Review Board.

### Knockdown of vimentin by shRNA

Vimentin knockdown was performed using MISSON lentiviral transduction particles (Sigma). Briefly, lentiviral transduction particles were produced from a lentiviral plasmid vector containing the shRNA sequences for the human vimentin gene: 3′-CCGGCGCCATCAACACCGAGTTCAACTCGAGTTGAACTCGGTGTTGATGGCGTTTTT-5′. The non-target shRNA control transduction particles containing the sequence 5′-CCGGCAACAAGATGAAGAGCACCAACTCGAGTTGGT GCTCTTCATCTTGTTGTTTTT-3′ do not target any human genes but activate the RNAi pathway. 40 ul of lentiviral particles were added to 1 × 10^5^ HPSE-high CAG myeloma cells and incubated for 18–20 h at 37°C in a humidified incubator. The next day, the medium containing the lentiviral particles was removed, and fresh complete RPMI medium added. The cells were then selected using puromycin (20 ug/ml) and assessed for vimentin knockdown by western blotting.

### Animal models and *in vivo* experiments

Five- to six-week-old male CB.17 SCID/SCID mice (Harlan-Sprague Dawley) were housed in individual cages (5 per cage) in a temperature (22°C) and humidity (50%) controlled room maintaining a 12 h light/12 h dark cycle. All experimental procedures and protocols were approved by the University of Alabama at Birmingham (UAB) Institutional Animal Care and Use Committee.

SCID subcutaneous (SCID-s.c.) model of MM. 1 × 10^6^ of HPSE-low or HPSE-high CAG cells were injected subcutaneously into the left flank of CB.17 SCID/SCID mice (8 mice injected HPSE-low cells, 16 mice injected HPSE-high cells). Alzet osmotic pumps (Durect Corporation) containing heparanase inhibitor SST0001 were inserted into the right flank [16] of 8 mice bearing CAG HPSE tumors 10 days after tumor injection. The pumps constantly released HPSE inhibitor SST0001 for 28 days at a dose of 30 mg/kg/day. Eight mice bearing HPSE-high tumors and 8 mice bearing HPSE-low tumors had Alzet osmotic pumps inserted that delivered PBS as SST0001 controls. After 28 days of treatment, all animals were euthanized; the tumors were collected, paraffin-embedded and sectioned for immunochemical staining and analysis.

Bone homing of MM cells. 1 × 10^6^ NT control or vimentin knockdown HPSE-high CAG-luc cells were inoculated into CB.17 SCID/SCID mice via tail vein (*n* = 5). The animals were imaged on an IVIS-100 system (Xenogen Corporation) bi-weekly after injection of tumor cells. All animals were euthanized at week 6 and sera collected for measurement of circulating immunoglobulin kappa light chain.

### Quantification of human immunoglobulin kappa light chain

The levels of human immunoglobulin kappa light chain in murine sera were measured to assess whole animal tumor burden. Sera collected during animal studies were stored at −80°C and analyzed by ELISA (Bethyl Laboratories) in duplicate as previously described [34]. The standard curve was linear between 15.6 and 1000 ng/ml, and samples were diluted to concentrations within this range.

### Cell spreading assay

96-well plates coated with fibronectin (50 ug/ml) were treated with 1% BSA in serum-free RPMI medium for 1 h at room temperature. After three washes, 1 × 10^4^ cells were added to each well and incubated in serum-free RPMI medium at 37°C in 5% CO_2_. Analysis of cell spreading was performed at 1 and 3 hr after cell plating and was based on assessment of cell morphology. Cells that assumed a long and thin morphology were considered spread, whereas non-spread cells retained a round shape with an uneven outline. The number of spread cells and total cells were enumerated in 5 random fields at 200×magnification for each sample at each time point. The cell spreading ratio was calculated by measuring the percentage of spreading cells in the number of total cells.

### MTT and cell migration assay

Cell proliferation was determined using a 3 [4,5-dimethylthiazol-2-y] -2, 5-diphenyltetrazolium (MTT) assay kit (Abnova) according to manufacturer instructions. Assays were performed at 24 and 48 h and each sample was assayed in triplicate.

HPSE-high and HPSE-high/Vim k/d cell mobility was determined using a commercially available migration assay (BD Biosciences). Briefly, 2 × 10^5^ cells in 500 μl serum-free medium were added in triplicate into inserts containing 8 μm pores and allowed to migrate towards complete medium in the bottom wells at 37°C and 5% CO_2_ for 48 hours. Cells that invaded into bottom wells were enumerated in triplicate, using a Z1 Dual threshold Coulter Counter (Beckman Coulter) [35].

### Statistical analysis

Statistical comparisons between two experimental groups were analyzed by Student's *t* test. For comparisons among multiple groups, ANOVA followed by a *post-hoc* Bonferroni correction was used. Immunohistochemical staining results were analyzed using Mann-Whitney *U* test. The correlations between heparanase and vimentin, and heparanase and fibronectin expression in MM patients' samples were assessed using Spearman correlation coefficient. *p* < 0.05 was considered statistically significant and is reported as such. Data are presented as Mean ± SD or SEM as indicated.

## SUPPLEMENTARY MATERIALS TABLES


