# Supplementary material for: Heparanase promotes myeloma progression by inducing mesenchymal features and motility of myeloma cells
Source: Oncotarget. 2016 Feb 3;7(10):11299–309. doi: 10.18632/oncotarget.7170 (PMC4905474; doi:10.18632/oncotarget.7170)
Supplement: Supplementary file 1 [file oncotarget-07-11299-s001.pdf]

Heparanase promotes myeloma progression by inducing mesenchymal features and motility of myeloma cells

Supplementary Materials

Supplementary Table S1: Heparanase and vimentin expression scores in myeloma cells within the bone marrow of 35 myeloma patients

Patient ID	Heparanase	Vimentin*
1	4	4
2	4	3
3	4	4
4	2	2
5	3	4
6	4	4
7	3	3
8	4	3
9	4	2
10	3	3
11	3	2
12	4	2
13	2	3
14	3	3
15	2	2
16	4	4
17	4	3
18	2	3
19	2	2
20	4	4
21	3	3
22	2	4
23	3	2
24	4	4
25	2	2
26	2	2
27	4	4
28	3	2
29	2	3
30	3	3
31	3	4
32	2	3

33	4	3
34	4	3
35	3	2

*Heparanasevs.vimentin spearman's correlation coefficient $r_s = 0.414$, $p = 0.014$.
Intensity was scored by two independent readers. Scored from 1 to 4 with 4 being the strongest intensity of staining observed. The correlation between the scores of staining intensity of heparanase and vimentin was calculated as a Spearman correlation coefficient. Expression of heparanase was significantly and positively correlated with vimentin ($r_s = 0.414$, $p = 0.014$).

Supplementary Table S2: Heparanase and fibronectin expression scores in myeloma cells within the bone marrow of myeloma patients

Patient ID	Heparanase	Fibronectin
1	2	2
2	2	2
3	2	2
4	2	2
5	2	1
6	2	1
7	2	2
8	3	2
9	3	3
10	4	4
11	4	3
12	4	3
13	4	2
14	4	4

Intensity was scored by two independent readers. Scored from 1 to 4 with 4 being the strongest intensity of staining observed. The correlation between the scores of staining intensity of heparanase and fibronectin was calculated asa Spearman correlation coefficient. Expression of heparanase was significantly and positively correlated with fibronectin ($r_s = 0.55$, $p < 0.05$).
